# Inhibition of Fatty Acid Oxidation as a New Target To Treat Primary Amoebic Meningoencephalitis

**DOI:** 10.1128/AAC.00344-20

**Published:** 2020-07-22

**Authors:** Maarten J. Sarink, Aloysius G. M. Tielens, Annelies Verbon, Robert Sutak, Jaap J. van Hellemond

**Affiliations:** a Department of Medical Microbiology and Infectious Diseases, Erasmus MC University Medical Center Rotterdam, Rotterdam, Netherlands; b Department of Biochemistry and Cell Biology, Faculty of Veterinary Medicine, Utrecht University, Utrecht, Netherlands; c Department of Parasitology, Faculty of Science, Charles University, BIOCEV, Vestec, Czech Republic

**Keywords:** *Naegleria fowleri*, *Naegleria gruberi*, drug targets, energy metabolism, lipid metabolism, therapy, thioridazine, treatment

## Abstract

Primary amoebic meningoencephalitis (PAM) is a rapidly fatal infection caused by the free-living amoeba Naegleria fowleri. The amoeba migrates along the olfactory nerve to the brain, resulting in seizures, coma, and, eventually, death. Previous research has shown that Naegleria gruberi, a close relative of N. fowleri, prefers lipids over glucose as an energy source. Therefore, we tested several already-approved inhibitors of fatty acid oxidation alongside the currently used drugs amphotericin B and miltefosine. Our data demonstrate that etomoxir, orlistat, perhexiline, thioridazine, and valproic acid inhibited growth of N. gruberi. We then tested these compounds on N. fowleri and found etomoxir, perhexiline, and thioridazine to be effective growth inhibitors. Hence, not only are lipids the preferred food source for N. gruberi, but also oxidation of fatty acids seems to be essential for growth of N. fowleri. Inhibition of fatty acid oxidation could result in new treatment options, as thioridazine inhibits N. fowleri growth in concentrations that can be reached at the site of infection. It could also potentiate currently used therapy, as checkerboard assays revealed synergy between miltefosine and etomoxir. Animal testing should be performed to confirm the added value of these inhibitors. Although the development of new drugs and randomized controlled trials for this rare disease are nearly impossible, inhibition of fatty acid oxidation seems a promising strategy as we showed effectivity of several drugs that are or have been in use and that thus could be repurposed to treat PAM in the future.

## Abstract (precis)

Primary amoebic meningoencephalitis (PAM) is a rapidly fatal infection caused by the free-living amoeba Naegleria fowleri. The amoeba migrates along the olfactory nerve to the brain, resulting in seizures, coma, and, eventually, death. Previous research has shown that Naegleria gruberi, a close relative of N. fowleri, prefers lipids over glucose as an energy source. Therefore, we tested several already-approved inhibitors of fatty acid oxidation alongside the currently used drugs amphotericin B and miltefosine.

## INTRODUCTION

The amoeba Naegleria fowleri causes primary amoebic meningoencephalitis (PAM), a rapidly fatal disease of the central nervous system (CNS) (1–3). N. fowleri is one of the three most common free-living amoebae that can infect humans, the others being *Acanthamoeba* spp. and Balamuthia mandrillaris. These amoebae are ubiquitously present, with N. fowleri reported on all continents, except Antarctica (4). In the United States, N. fowleri infections occur mostly in healthy children and young adults during recreational water activities, such as swimming, diving, and rafting (5–7). In the Indian subcontinent, the correlation with age is less clear, probably because ablution rituals, washing, and a lack of chlorination play a large role in the epidemiology (7, 8). When water containing N. fowleri makes contact with the nasal epithelium, the trophozoite stage of the amoeba can migrate along the olfactory nerve, through the cribriform plate to the olfactory bulb within the CNS (2, 3, 9). Once inside the brain, the trophozoites cause necrosis and acute inflammation, ultimately leading to death in over 95% of the cases (1, 3). There is concern that global warming and changes in the ecosystems that N. fowleri inhabits may lead to more cases worldwide (7, 8, 10). A wide range of antifungals and antibiotics have been used to treat PAM with various degrees of effectivity. Most evidence is available for amphotericin B and miltefosine, but CNS penetration of these drugs is poor (11–14). Because of the high mortality rate, more-effective drugs are urgently needed (15).

Inhibition of metabolic processes essential to microorganisms is a fruitful strategy for the development of effective drugs (16). Several widely used drugs, such as the antimalarials atovaquone and proguanil and the broad-spectrum antiprotozoal, antihelminthic, and antiviral drug nitazoxanide, target the metabolism of the pathogen to exert their killing effect (17, 18).

**FIG 1:**
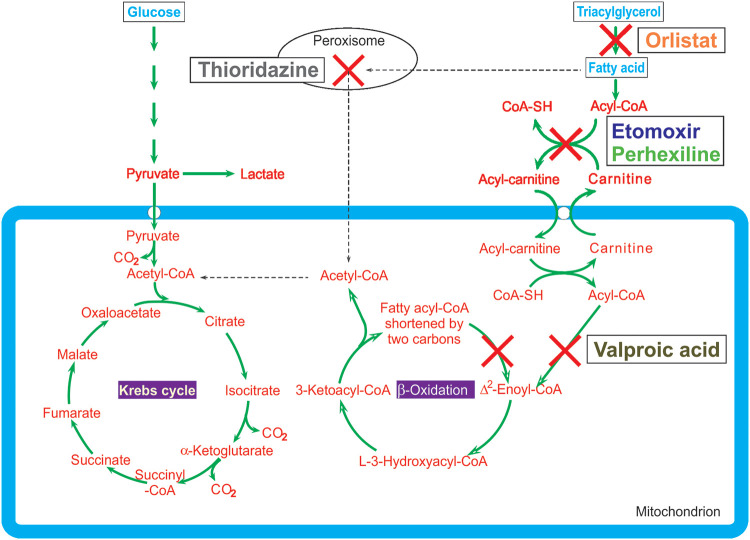
Main pathways of energy metabolism of N. gruberi with targets of different fatty acid oxidation inhibitors depicted as crosses. Dashed lines indicate uncertainties of the actual processes. CoA, coenzyme A.

Previous research by our group showed that N. gruberi, a close relative of N. fowleri, prefers fatty acids as a food source (19). This led us to the hypothesis that inhibiting fatty acid oxidation (FAO) could inhibit growth of or even kill the amoeba. We identified several drugs that are currently used or have been used to inhibit fatty acid metabolism in different parts of this pathway. All of those drugs target enzymes that are present in the N. gruberi and N. fowleri genome (19) (see also Fig. 1 and Discussion). As the fatty acid preference was shown in N. gruberi, we first determined the effects of these compounds on N. gruberi. We then tested promising compounds on the actual pathogen, N. fowleri, and finally determined whether there was synergy present when the compounds were combined in a checkerboard assay.

## RESULTS

**FIG 2:**
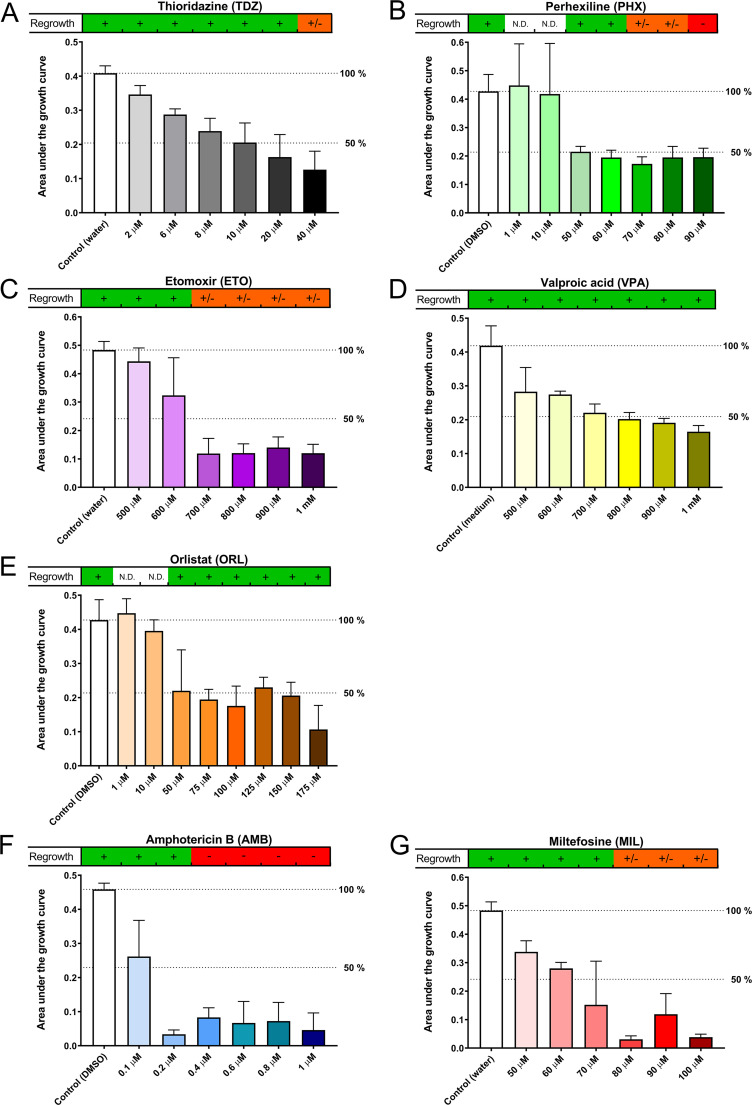
Growth curves of Naegleria gruberi were obtained in the presence or absence of inhibitors of fatty acid oxidation or drugs currently used to treat primary amoebic meningoencephalitis. Optical density was measured daily over a 5-day period. Data shown represent the area under the growth curve (AUC) determined for the indicated compounds and the respective controls, including lines representing 100% and 50% of the control AUC. At the top of the graph, data representing the capacity for regrowth are shown. +, clear regrowth; +/−, inconsistent or little regrowth; −, never any regrowth; N.D., not done. Experiments were performed twice in triplicate wells; error bars represent standard deviations (SD).

The inhibitory effects of all compounds on N. gruberi determined through area under the curve (AUC) calculation are represented in Fig. 2. Thioridazine (TDZ) inhibited growth of N. gruberi in a concentration-dependent manner, inhibiting 50% of growth at approximately 10 μM (Fig. 2A). Addition of perhexiline (PHX) resulted in an inhibition level of about 50% at concentrations above 50 μM (Fig. 2B). Etomoxir (ETO) addition resulted in clear inhibition at concentrations above 600 μM (Fig. 2C), and addition of valproic acid (VPA) resulted in inhibition of growth in a concentration-dependent manner, with inhibition of 50% of growth at around 700 μM (Fig. 2D). Orlistat (ORL) inhibited circa 50% of growth at concentrations of 50 μM and higher (Fig. 2E), and amphotericin B (AMB) was very effective at inhibiting growth, inhibiting circa 75% at concentrations of 0.2 μM and higher (Fig. 2F). Addition of miltefosine (MIL) resulted in inhibition in a concentration-dependent manner, with efficient inhibition of growth at 80 μM (Fig. 2G). The capacities for regrowth of the amoebae after 5 days of exposure differed for the examined compounds, as can be seen in the bars above the individual graphs in Fig. 2. Amoebae incubated with VPA and ORL showed regrowth for all concentrations used, while PHX consistently prevented regrowth at 90 μM. The amoebae showed regrowth after TDZ exposure at up to a concentration of 20 μM, while the amoebae showed regrowth after ETO exposure at up to a concentration of 600 μM. Amoebae incubated with MIL showed regrowth after exposure to concentrations below 80 μM and inconsistent regrowth at the examined concentrations over 80 μM. AMB was most effective in preventing regrowth, always blocking regrowth at a concentration of 0.4 μM or higher (Fig. 2).

**FIG 3:**
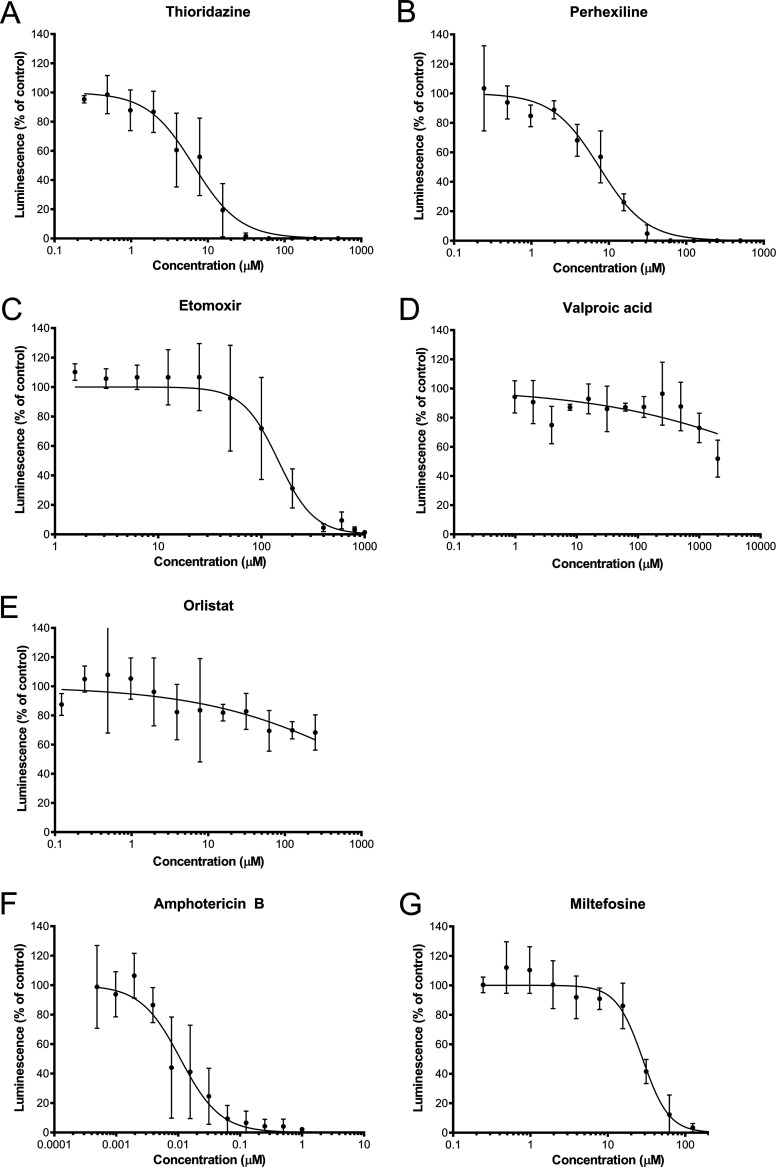
Luminescence as a percentage of control after compound exposure to Naegleria fowleri for 24 h in 2-fold serial dilutions. Luminescence was measured after addition of CellTiter-GLO ATP stain, in the presence or absence of inhibitors of fatty acid oxidation or drugs currently used to treat primary amoebic meningoencephalitis. Experiments were performed in triplicate; error bars represent SD.

**FIG 4:**
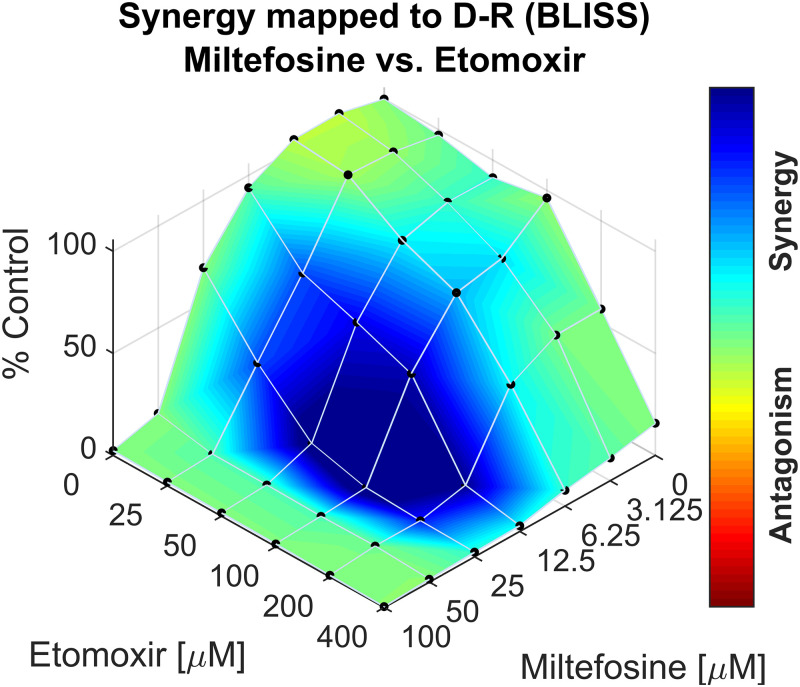
Surface response plot of checkerboard assay of concentrations of etomoxir and miltefosine against Naegleria fowleri using the BLISS model. Etomoxir and miltefosine were separately tested and combined in 5 and 6 concentrations, respectively. Luminescence was measured after 24 h of exposure and after addition of CellTiter-GLO ATP stain. Raw luminescence data were normalized as a percentage of the control, results were analyzed, and the plot was generated with the combenefit program. Colors indicate presence or absence of synergy.

Next, the compounds were also assessed for their effect on N. fowleri. This was done in two ways: via viability staining with CellTiter-GLO and with direct cell counting using a flow cytometer. The 50% inhibitory (IC_50_) concentrations of all drugs on both organisms are listed in Table 1. The IC_50_ results reported here for compounds tested on N. gruberi represent approximations, as the range of concentrations tested was narrow. Results of the CellTiter-GLO viability stain assay can be seen in Fig. 3, and results of cell counting can be seen in Fig. S1 in the supplemental material. These figures show that absence of viability and absence of cell growth were observed after exposure to TDZ, PHX, and ETO, revealing that these compounds are effective against N. fowleri as well as against N. gruberi. The efficacy of ETO and PHX was higher against N. fowleri than against N. gruberi, and the IC_50_ levels of both drugs were about 5-fold lower against N. fowleri than against N. gruberi. VPA and ORL showed some inhibition of N. fowleri growth at high concentrations, but their efficacy was much lower against N. fowleri than against N. gruberi. The effects of AMB and MIL were roughly similar against N. gruberi and N. fowleri. The IC_50_ calculations show concordance between the two methods, confirming the efficacy of the compounds against N. fowleri in two ways (Table 1). Next, compounds were combined in these IC_50_ concentrations to screen for a possible synergistic effect of combinations of compounds against N. fowleri (see Table S1 in the supplemental material). Checkerboard assays were performed for the best six combinations of the drugs, after which *F*_min_ was calculated. The combinations MIL plus PHX, MIL plus TDZ, MIL plus VPA, PHX plus TDZ, and TDZ plus VPA showed additivity. The combination of ETO and MIL resulted in an F of 0.5, indicating that synergy was present when these drugs were combined (Table S1). Further analysis of the synergistic effect of the combination ETO and MIL with the program Combenefit resulted in a mapped surface analysis whose results can be seen in Fig. 4. The map shows that the synergy was most pronounced when 12.5 and 25 μM concentrations of MIL were combined with 25 to 200 μM concentrations of ETO.

**TABLE 1:** IC_50_ values determined for compounds tested on Naegleria gruberi and N. fowleri^*a*^

Compound	Target	Naegleria gruberi		Naegleria fowleri			
		IC_50_ (μM) (area under the growth curve)	95% CI	IC_50_ (μM) (CellTiter- GLO)	95% CI	IC_50_ (μM) (cell counting)	95% CI
Thioridazine	Peroxisomal lipid oxidation (26)	13	(10.6–16.0)	6.5	(5.0–8.4)	9.8	(7.3–12.9)
Perhexiline	CPT-1 (28)	56	(46.6–65.3)	7.5	(6.0–9.4)	17.4	(14.9–20.4)
Etomoxir	CPT-1 (29)	666	(625–708)	146.0	(114.9–185.5)	108.7	(78.2–148)
Valproic acid	Acyl-CoA dehydrogenase (30)	788	(741–845)	—^*b*^		—	
Orlistat	Lipase (27)	75	(56.1–98.2)	—		—	
Amphotericin B	Sterols (15)	0.09	(0.04–0.13)	0.011	(0.007–0.016)	0.027	(0.016–0.044)
Miltefosine	Unknown (43)	61	(56.5–64.7)	28.2	(23.8–33.4)	33.4	(25.6–43.0)

a *N. gruberi* growth curve data were obtained by measuring optical density daily over a 5-day period, after which area under the growth curve values were calculated. The IC_50_ data from compounds tested on *N. gruberi* represent approximations, as the range of concentrations tested was narrow. N. fowleri was tested in two ways: with CellTiter-GLO ATP stain and through cell counting with a guava EasyCyte flow cytometer. Levels of CellTiter-GLO luminescence were determined after 24 h of incubation, and cell counts were determined after 72 h of incubation. Raw data were normalized as a percentage of the levels measured for the respective controls. Nonlinear regression was performed by the use of GraphPad Prism 8 as [inhibitor] versus normalized response with a variable slope, after which the IC_50_ data were calculated. Acyl-CoA, acyl-coenzyme A; CI, confidence interval.

b —, calculation not possible.

## DISCUSSION

Our study showed that fatty acid oxidation (FAO) inhibitors clearly inhibited growth of both N. gruberi and N. fowleri *in vitro*. Hence, not only are lipids the preferred food source for N. gruberi, but oxidation of fatty acids seems to be essential also for growth of N. fowleri. The current treatment regimen using miltefosine (MIL) and amphotericin B (AMB) was confirmed to be effective at inhibiting growth *in vitro*, which is in agreement with previous reports (20–23) and validates our assays performed to detect compounds that inhibit growth of *Naegleria*. The importance of fatty acid oxidation for N. fowleri was demonstrated in a recent *in vivo* study, as Herman et al. observed upregulation of genes of N. fowleri involved in FAO after mouse passage (24). Our results now show that FAO inhibition is a valid target for new PAM therapy options, as etomoxir (ETO), perhexiline (PHX), and thioridazine (TDZ) showed total growth inhibition of N. fowleri. Furthermore, our results show that there is additivity of MIL combined with PHX, TDZ, and valproic acid (VPA) and synergy between ETO and MIL, providing evidence that inhibition of fatty acid oxidation can be a valuable addition to the current treatment regimen.

We observed some differences between N. gruberi and N. fowleri in the levels of efficacy of the FAO inhibitors. All FAO inhibitors affected N. gruberi growth, but the effects of VPA and orlistat (ORL) were much less profound on N. fowleri than on N. gruberi. In contrast, ETO and PHX were more effective at inhibiting growth of N. fowleri than N. gruberi. Taking the targets of the FAO inhibitors into account, we can hypothesize on the reasons for these differences. The investigated FAO inhibitors affect different enzymes involved in lipid metabolism (depicted in Fig. 1). TDZ inhibits peroxisomal oxidation of lipids (25, 26). ORL inhibits lipases, enzymes that hydrolyze triacylglycerol, thereby obstructing the first step in the breakdown of lipids (27). ETO and PHX inhibit carnitine palmitoyltransferase-1 (CPT-1), blocking transport of fatty acids into mitochondria (28, 29). Among other activities, VPA interferes mainly with mitochondrial β-oxidation (30). The targets of the FAO inhibitors are present in the N. gruberi genome as well as in the N. fowleri genome, showing that regarding the metabolic properties, the two organisms are very much alike (19, 31, 32). However, this does not imply that the enzymes are exactly identical in amino acid sequence. Minor amino acid differences could result in small structural differences and hence in differences in the effects of the various drugs. Furthermore, unavoidable differences between N. fowleri and N. gruberi under *in vitro* growth conditions could play a role, as the optimal culture media (PYNFH versus Bacto Casitone) and culture temperatures (25°C versus 37°C) are different between the two, resulting in different metabolic rates.

We observed that several FAO inhibitors show additivity (PHX, TDZ, and VPA) or synergy (ETO) when combined with MIL. Unfortunately, synergy between AMB and the FAO inhibitors was not observed. This would be of importance, as AMB has serious side effects (14). There is also a risk for serious adverse events when using MIL (33). We did observe synergy between ETO and MIL, which is a promising result as this could potentially lead to lowering of MIL dosages and subsequent reduction in the risk of serious adverse events. ETO has been in use for some time but was retired due to its adverse side effects in the liver. Currently, it is being repurposed as an anticancer agent (34). Unfortunately, there are no data on the pharmacokinetics of ETO, so we do not know the clinical applicability of ETO. We found a relatively high IC_50_ against N. fowleri of approximately 100 to 150 μM, but the synergy shown with MIL deserves further investigation in an animal model.

Of all FAO inhibitors tested, TDZ showed the lowest IC_50_ (approximately 6 to 10 μM) against N. fowleri in our study. TDZ has been in use as an antipsychotic drug since the early 1950s and was originally identified as a dopamine receptor 2 antagonist. Later, TDZ was also shown to be a selective inhibitor of peroxisomal β-oxidation (25, 26). It is now being repurposed as an anticancer, anti-inflammatory, and antimicrobial agent (35–38). The pharmacokinetics of TDZ are well studied. In a recent clinical study, the sum of TDZ and its metabolites in serum approached 10 μM (37). Furthermore, TDZ has been shown to accumulate in brain tissue of chronically treated patients, resulting in concentrations 10-fold higher than that in serum (39). Although AMB is effective at nanomolar concentrations, AMB and MIL are known to have poor CNS penetration (11–14). This could possibly explain the poor prospects for treatment of patients with PAM and emphasizes the possible benefit of TDZ.

Development and testing of new drugs for PAM are difficult, as randomized controlled trials for the treatment of PAM are impossible due to the rapidly fatal nature of the disease and its relatively rare occurrence. Repurposing existing drugs is therefore the most promising way to obtain additional drugs to combat PAM. There are numerous examples of drugs that have been successfully repurposed to treat rare diseases (40). All tested FAO inhibitors (including TDZ and ETO) have been or are still in clinical use. TDZ inhibits N. fowleri growth at concentrations that can be reached at the site of infection, and checkerboard assays revealed synergy between MIL and ETO. Further animal testing should be performed to confirm the added value of these inhibitors.

## MATERIALS AND METHODS

### Chemicals and amoeba culture.

N. gruberi strain NEG-M (ATCC 30224) was grown axenically at 25°C in modified PYNFH medium (peptone, yeast extract, yeast nucleic acid, folic acid, 10% heat-inactivated fetal bovine serum, 100 units/ml penicillin, 100 μg/ml streptomycin, and 40 μg/ml gentamicin) (ATCC medium 1034), as described before (19). N. fowleri strain HB-1, kindly provided by Hana Pecková (Institute of Parasitology, Biology Center CAS, Czech Republic), was grown axenically at 37°C in Bacto Casitone medium. Experiments with N. fowleri were conducted at biosafety level 2 (BSL 2) according to the ATCC guidelines and as specified by the Charles University guidelines. Bacto Casitone medium is composed of 2% Bacto Casitone, 10% heat-inactivated fetal bovine serum, penicillin (100 U/ml), and streptomycin (100 μg/ml). All experiments were performed using trophozoites harvested during logarithmic-phase growth by repeatedly tapping cell culture flasks containing amoebae to detach trophozoites. Amphotericin B (AMB), etomoxir (ETO), miltefosine (MIL), thioridazine (TDZ), orlistat (ORL), perhexiline (PHX), and valproic acid (VPA) were purchased from Sigma. CellTiter-Glo 2.0 Cell viability assay was purchased from Promega. Translucent flat-bottom 96-well plates were purchased from Greiner Bio-One. Black flat-bottom 96-well plates were purchased from Thermo Fisher Scientific.

### Inhibition assays for N. gruberi.

To screen the effects of fatty acid oxidation inhibitors and current therapies for PAM on N. gruberi, we determined compound efficacy with optical density (OD) measurements. Drugs were prepared as stock solutions as follows: TDZ, 10 mM in water; PHX, 50 mM in dimethyl sulfoxide (DMSO); ETO, 10 mM in water; VPA, 20 mM in water; ORL, 35 mM in DMSO; AMB, 10 mM in DMSO; MIL, 10 mM in water. Stock solutions were diluted in water or PYNFH medium and added as a volume of 10 μl to 1 × 10^4^ N. gruberi trophozoites in 90 μl of PYNFH. Compounds were tested per plate in triplicate in at least two independent experiments; controls contained equivalent concentrations of compound solvents (water, PYNFH or DMSO). Plates were incubated at 25°C, and OD measurements of the contents of the 96 wells were performed every 24 h using a FLUOstar Optima microplate reader. Regrowth capacity was assessed by collecting the whole contents of the wells at day 5 by vigorous pipetting, after which the content (100 μl) was added to Eppendorf tubes containing 1 ml PYNFH medium. Samples were washed by centrifugation at 1,000 relative centrifugal force (rcf) and subsequent careful replacement of the supernatant with fresh PYNFH medium. After this washing cycle was repeated three times, 1 ml supernatant was discarded and the remaining contents were added to a new plate. Controls were diluted 10× after the washing step to allow proper detection of regrowth in these samples. OD was measured for a further period of 9 days of incubation at 25°C.

### Inhibition assays for N. fowleri.

For N. fowleri, two independent methods were used to determine compound efficacy: a CellTiter-GLO luminescence-based ATP stain method (less sensitive to the number of amoeba but more sensitive to viability) and cell counting by flow cytometry (sensitive to the number of amoeba but with count determined irrespective of viability). Similar stock solutions were prepared for N. fowleri experiments as those used for N. gruberi experiments. For CellTiter-GLO experiments, stock solutions were diluted and added to a black 96-well plate as 10 μl compound–80 μl Bacto Casitone, after which 300 N. fowleri cells in 10 μl Bacto Casitone were added to each well for a total volume of 100 μl. After 24 h of incubation at 37°C, CellTiter-GLO reagent was added and luminescence was determined by the use of a CLARIOstar microplate reader. For flow cytometry experiments, stock solutions were diluted and added to translucent 96-well plate wells as 20 μl compound–178 μl Bacto Casitone, after which 60 N. fowleri cells in 2 μl Bacto Casitone were added to each well for a total volume of 200 μl. After 72 h of incubation at 37°C, paraformaldehyde was added to obtain a 1.5% concentration, after which cell counting was performed by the use of a Guava EasyCyte flow cytometer. Appropriate gating was applied to all samples. All experiments were performed in triplicate; all plates contained positive controls in triplicate wells with equivalent concentrations of compound solvents (water, Bacto Casitone, or DMSO) and negative controls without amoebae.

### Checkerboards assay.

Twofold dilutions were prepared of MIL (100 to 3.13 μM), ETO (400 to 25 μM), PHX (25 to 1.61 μM), TDZ (25 to 0.8 μM), and VPA (2,000 to 62.5 μM). Drugs were added to black 96-well plates in a 5-by-6 checkerboard design as described before (41). The 96-well plates were inoculated with 300 N. fowleri cells in Bacto Casitone per well. After 24 h of incubation at 37°C, CellTiter-GLO reagent was added and luminescence was determined by the use of a CLARIOstar microplate reader. All experiments were performed in triplicate; all plates contained positive controls in triplicate wells with equivalent concentrations of compound solvents (water, Bacto Casitone, or DMSO) and negative controls without amoebae.

### Data analysis.

GraphPad Prism 8 was used to process data. For N. gruberi, graphs of separate wells were constructed with OD values represented on the *y* axis and time (in days) on the *x* axis. Area under the curve (AUC) was then calculated by the use of GraphPad Prism 8, and the calculated data were combined into a bar chart. To determine IC_50_ values, results were normalized and nonlinear regression with variable slope was performed. For N. fowleri, luminescence data and cell counts were normalized as a percentage of the respective control, after which nonlinear regression with variable slope was performed to determine IC_50_ values. To determine synergy, analysis was performed using the fractional inhibitory concentration index (FIC_i_), as described before (41). Briefly, FIC_i_ values were determined for the wells with the lowest concentration of drugs that resulted in <10% luminescence of the growth control without drugs. FIC_i_ was calculated as $\text{ΣFIC}=\frac{C_{\text{a}}}{{\text{MIC}}_{\text{a}}}+\frac{C_{\text{b}}}{{\text{MIC}}_{\text{b}}}$, with *C*_a_ and *C*_b_ being the concentrations of the drugs in the well and MIC_a_ and MIC_b_ the lowest concentrations of separate drugs that resulted in <10% of luminescence. *F*_min_ was defined as the lowest ∑FIC_i_ value. Additivity was determined as 0.5 < ∑FIC_i_ < 2, synergy as ∑FIC_i_ = ≤0.5, and antagonism as ∑FIC_i_ = >2. Surface response analysis was performed with the Combenefit program (42).

## Supplementary Material

Supplemental file 1
