# Supplementary material for: Inhibition of Fatty Acid Oxidation as a New Target To Treat Primary Amoebic Meningoencephalitis
Source: Antimicrob Agents Chemother. 2020 Jul 22;64(8):e00344-20. doi: 10.1128/AAC.00344-20 (PMC7526813; doi:10.1128/AAC.00344-20)
Supplement: Supplemental file 1 [file AAC.00344-20-s0001.pdf]

Supplementary Data

Supplementary Table S1:

Combination	Percentage of separate drug #1	Percentage of separate drug #2	Average	Further checkerboards?	F_{min} of checkerboard	Interpretation
ETO + MIL	2.8	3.8	3.3	YES	0.5	Synergy
ETO + PHX	66.7	84.1	75.4	NO		
ETO + AMB	93.8	77.5	85.7	NO		
ETO + ORL	113.2	131.4	122.3	NO		
ETO + TDZ	63.0	107.1	85.1	NO		
ETO + VPA	74.6	82.0	78.3	NO		
MIL + PHX	56.8	52.7	54.7	YES	1.0625	Additivity
MIL + AMB	118.1	70.3	94.2	NO		
MIL + ORL	74.8	62.6	68.7	NO		
MIL + TDZ	42.7	53.2	48.0	YES	1.0312	Additivity
MIL + VPA	74.6	60.1	67.4	YES	1.0156	Additivity
PHX + AMB	96.0	62.3	79.2	NO		
PHX + ORL	77.4	71.1	74.2	NO		
PHX + TDZ	46.7	63.1	54.9	YES	1.0	Additivity
PHX + VPA	79.7	69.3	74.5	NO		
AMB + ORL	81.1	115.2	98.2	NO		
AMB + TDZ	48.7	101.6	75.1	NO		
AMB + VPA	77.5	105.0	91.3	NO		
ORL + TDZ	62.8	92.9	77.9	NO		
ORL+ VPA	97.7	93.6	95.6	NO		
TDZ + VPA	80.2	51.7	65.9	YES	1.0	Additivity

Table S1:

Fatty acid oxidation inhibitors and drugs currently used to treat primary amoebic meningoencephalitis were added separately and combined in IC_{50} concentrations to Naegleria fowleri. Luminescence was measured at 24 hours after addition of CellTiter-GLO. Luminescence data of wells with combined drugs was normalised as a percentage of the luminescence of respective drugs tested separately. Experiments were performed in triplicate.

Supplementary Figure S1:

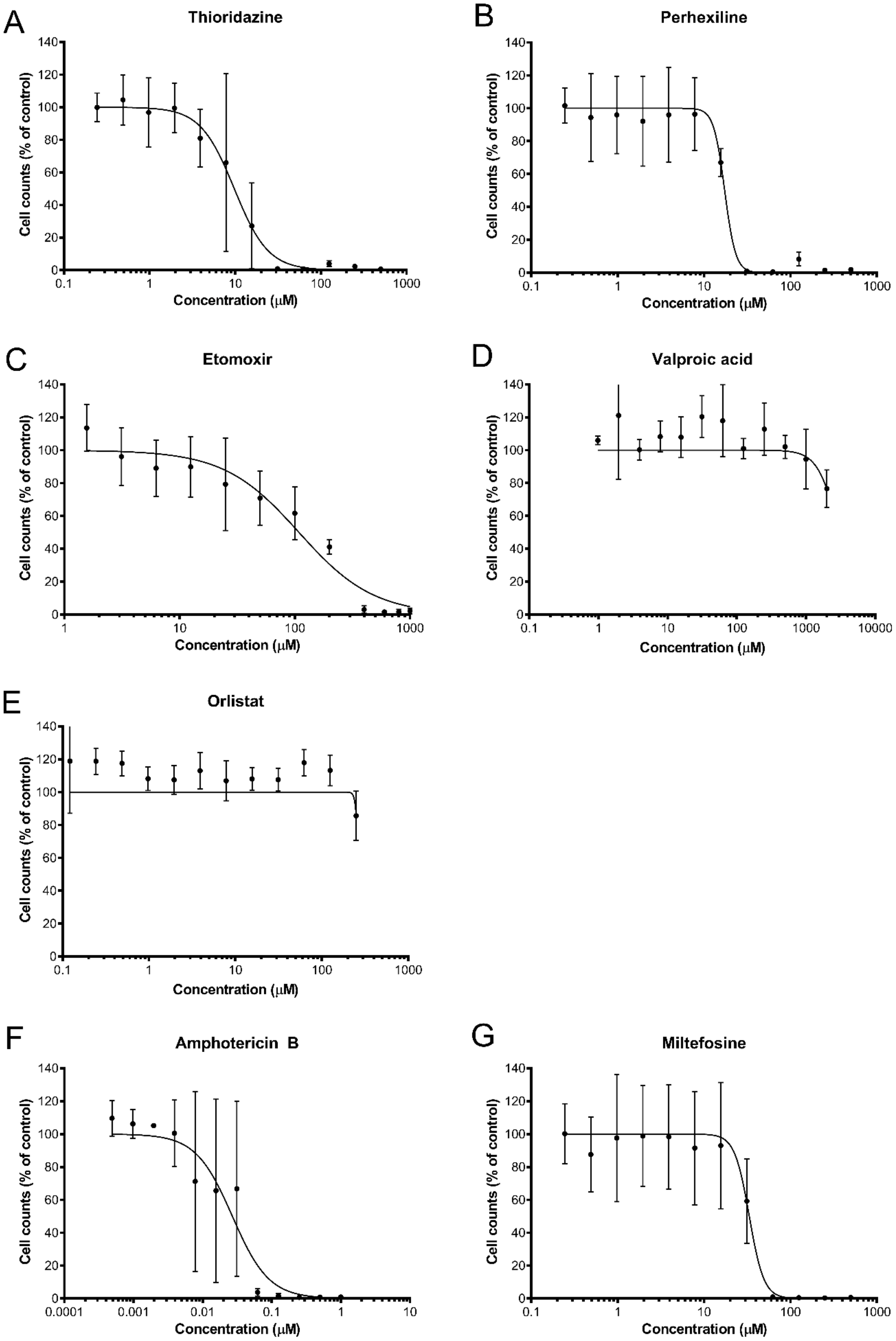

Figure S1:

Cell counts as a percentage of control after compound exposure to *Naegleria fowleri* for 72 hours in twofold serial dilutions. Cells were counted by guava EasyCyte flow cytometer in the presence or absence of inhibitors of fatty acid oxidation or drugs currently used to treat primary amoebic meningoencephalitis. Experiments were performed in triplicate, error bars are SD.
